# 2-(2-Hy­droxy­propan-2-yl)-6-(prop-2-yn­yloxy)-1-benzofuran-3(2*H*)-one

**DOI:** 10.1107/S1600536812045606

**Published:** 2012-11-17

**Authors:** Henok H. Kinfe, Yonas H. Belay, Zanele H. Phasha

**Affiliations:** a Research Center for Synthesis and Catalysis, Department of Chemistry, University of Johannesburg (APK Campus), PO Box 524, Auckland Park, Johannesburg, 2006, South Africa

## Abstract

In the title compound, C_14_H_14_O_4_, the prop-2-yn­yloxy O—C—C C plane [maximum deviation = 0.0116 (12) Å] forms a dihedral angle of 78.44 (9)° with the benzofuran-3(2*H*)-one ring system. In the crystal, mol­ecules are linked by O—H⋯O hydrogen bonds, forming a tape along the *a*-axis direction. C—H⋯O inter­actions are observed between the tapes.

## Related literature

For background to the development of hybrid drug candidates against tuberculosis, malaria and cancer, see: Morphy *et al.* (2004 ▶). For details of the synthesis of the title compound, see: Hoogendoorn *et al.* (2011 ▶).
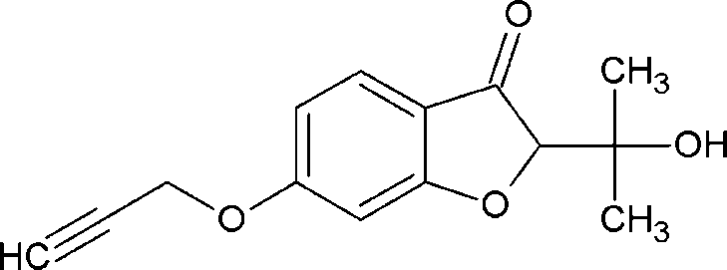


## Experimental

### 

#### Crystal data

C_14_H_14_O_4_
*M* *_r_* = 246.25
Triclinic, 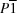

*a* = 5.4199 (2) Å
*b* = 9.0785 (3) Å
*c* = 12.3555 (4) Å
α = 85.758 (2)°
β = 80.455 (2)°
γ = 81.829 (2)°
*V* = 592.65 (4) Å^3^
*Z* = 2
Cu *K*α radiation
μ = 0.84 mm^−1^
*T* = 100 K
0.39 × 0.11 × 0.11 mm

#### Data collection

Bruker APEX DUO 4K CCD diffractometer
Absorption correction: multi-scan (*SADABS*; Bruker, 2008 ▶) *T*_min_ = 0.736, *T*_max_ = 0.913
10266 measured reflections
1983 independent reflections
1888 reflections with *I* > 2σ(*I*)
*R*_int_ = 0.025

#### Refinement

*R*[*F* ^2^ > 2σ(*F* ^2^)] = 0.032
*wR*(*F* ^2^) = 0.082
*S* = 1.03
1983 reflections
166 parameters
H-atom parameters constrained
Δρ_max_ = 0.21 e Å^−3^
Δρ_min_ = −0.17 e Å^−3^

### 

Data collection: *APEX2* (Bruker, 2011 ▶); cell refinement: *SAINT* (Bruker, 2008 ▶); data reduction: *SAINT*; program(s) used to solve structure: *SHELXS97* (Sheldrick, 2008 ▶); program(s) used to refine structure: *SHELXL97* (Sheldrick, 2008 ▶); molecular graphics: *ORTEP-3 for Windows* (Farrugia, 1997 ▶); software used to prepare material for publication: *WinGX* (Farrugia, 1999 ▶).

## Supplementary Material

Crystal structure: contains datablock(s) global, I. DOI: 10.1107/S1600536812045606/is5212sup1.cif

Structure factors: contains datablock(s) I. DOI: 10.1107/S1600536812045606/is5212Isup2.hkl

Supplementary material file. DOI: 10.1107/S1600536812045606/is5212Isup3.cml

Additional supplementary materials:  crystallographic information; 3D view; checkCIF report

## Figures and Tables

**Table 1:** Hydrogen-bond geometry (Å, °)

*D*—H⋯*A*	*D*—H	H⋯*A*	*D*⋯*A*	*D*—H⋯*A*
O1—H⋯O4^i^	0.84	2.01	2.8328 (12)	167
C1—H1⋯O1^ii^	0.95	2.45	3.3283 (16)	154
C5—H5⋯O1^iii^	0.95	2.52	3.3809 (15)	152

Symmetry codes: (i) 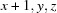; (ii) 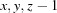; (iii) 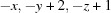.

**Fig. 1.**
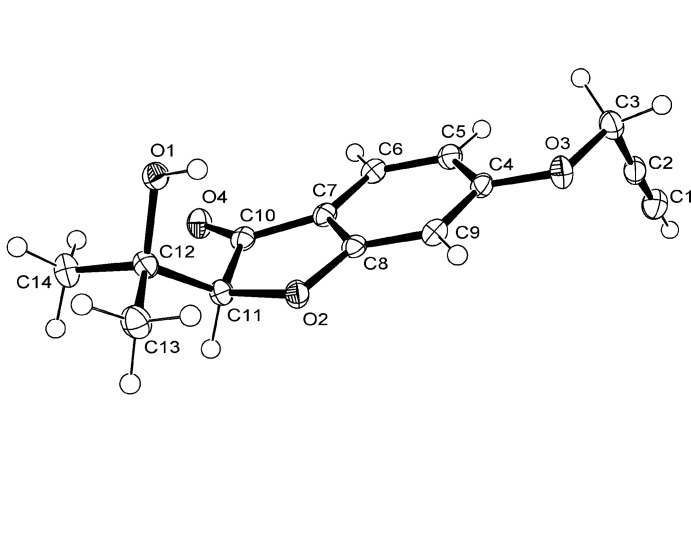
The molecular structure of the title compound with labeling and displacement ellipsoids drawn at the 50% probability level.

**Fig. 2.**
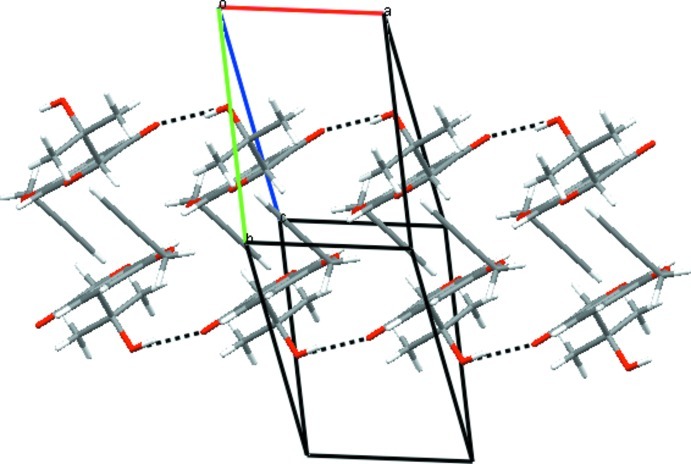
A portion of the crystal packing viewed approximately down the b axis. Dotted lines denote intermolecular O—H···O interactions.

## supplementary crystallographic information

### Comment

In our research in the development of hybrid drug candidates against tuberculosis, malaria and cancer (Morphy *et al.*, 2004), the title compound was synthesized as a building starting material. The title compound was synthesized by the reaction of 6-hydroxy-benzofuran-3-one with propargyl bromide in the presence of potassium carbonate at relatively high temperature (Hoogendoorn *et al.*, 2011). Herein we report the crystal structure of the title compound.

In the crystal structure of the title compound, the propyn-1-yloxy group (C1–C3/O3) forms a dihedral angle of 78.44 (9)° with the fused benzofuran-3-one ring system (Fig. 1). The crystal packing is stabilized by O—H···O interactions (Table 1 and Fig. 2). The C—H···O interactions are also observed (Table 1).

### Experimental

A solution of 6-hydroxy-benzofuran-3-one (1 g, 6.66 mmol) in dry acetone was treated with potassium carbonate (1.3 g, 9.32 mmol). The reaction mixture was heated at a temperature of 70–80 °C for about 30 minutes and then propargyl bromide (1.6 ml, 14.65 mmol) was added to it. The combined solution was stirred for about 2.5 h and concentrated under vacuum. The residue was diluted with water and extracted three times with ethyl acetate. The combined organic layer was washed with brine and water and dried over anhydrous magnesium sulfate. After that filtered and the filtrate solid product was recrystalized from ethyl acetate and hexane to afford 70% of the target compound as yellow crystal (m.p. 118–120 °C).

### Refinement

All hydrogen atoms were positioned in geometrically idealized positions with C—H = 1.00 Å (methine), 0.99 Å (methylene), 0.98 Å (methyl), 0.95 Å (aromatic), and 0.84 Å (hydroxyl). All hydrogen atoms were allowed to ride on their parent atoms with *U*_iso_(H) = 1.2*U*_eq_, except for the methyl and hydroxyl hydrogen atoms where *U*_iso_(H) = 1.5*U*_eq_ was utilized. The initial positions of methyl hydrogen atoms were located in a difference Fourier map and refined as a fixed rotor.

### Crystal data

**Table:**

C_14_H_14_O_4_	*Z* = 2
*M**_r_* = 246.25	*F*(000) = 260
Triclinic, *P*1	*D*_x_ = 1.38 Mg m^−^^3^
Hall symbol: -P 1	Cu *K*α radiation, λ = 1.54178 Å
*a* = 5.4199 (2) Å	Cell parameters from 4605 reflections
*b* = 9.0785 (3) Å	θ = 4.9–66.1°
*c* = 12.3555 (4) Å	µ = 0.84 mm^−^^1^
α = 85.758 (2)°	*T* = 100 K
β = 80.455 (2)°	Needle, colourless
γ = 81.829 (2)°	0.39 × 0.11 × 0.11 mm
*V* = 592.65 (4) Å^3^	

### Data collection

**Table:**

Bruker APEX DUO 4K CCD diffractometer	1983 independent reflections
Incoatec Quazar Multilayer Mirror monochromator	1888 reflections with *I* > 2σ(*I*)
Detector resolution: 8.4 pixels mm^-1^	*R*_int_ = 0.025
φ and ω scans	θ_max_ = 66.3°, θ_min_ = 4.9°
Absorption correction: multi-scan (*SADABS*; Bruker, 2008)	*h* = −4→6
*T*_min_ = 0.736, *T*_max_ = 0.913	*k* = −10→10
10266 measured reflections	*l* = −14→14

### Refinement

**Table:**

Refinement on *F*^2^	Primary atom site location: structure-invariant direct methods
Least-squares matrix: full	Secondary atom site location: difference Fourier map
*R*[*F*^2^ > 2σ(*F*^2^)] = 0.032	Hydrogen site location: inferred from neighbouring sites
*wR*(*F*^2^) = 0.082	H-atom parameters constrained
*S* = 1.03	*w* = 1/[σ^2^(*F*_o_^2^) + (0.0364*P*)^2^ + 0.2704*P*] where *P* = (*F*_o_^2^ + 2*F*_c_^2^)/3
1983 reflections	(Δ/σ)_max_ < 0.001
166 parameters	Δρ_max_ = 0.21 e Å^−^^3^
0 restraints	Δρ_min_ = −0.17 e Å^−^^3^

### Special details

**Table:**

Experimental. Analytical data: ^1^H NMR (CDCl_3_, 400 MHZ): δ 7.55 (d, 1H), 6.69 (s, 1H), 6.66 (s, 1H), 4.74 (s, 2H), 4.36 (s, 1H), 3.26 (s, 1H), 2.58 (s, 1H), 1.33 (s, 3H), 1.18 (s, 3H); ^13^C NMR (CDCl_3_, 400 MHZ): δ 199.0, 175.0, 166.2, 125.4, 115.5, 112.1, 97.4, 89.8, 72.3, 56.3, 25.9, 23.9.The intensity data was collected on a Bruker Apex DUO 4 K CCD diffractometer using an exposure time of 5 s/frame. A total of 4558 frames were collected with a frame width of 1° covering up to θ = 66.28° with 95.2% completeness accomplished.
Geometry. All s.u.'s (except the s.u. in the dihedral angle between two l.s. planes) are estimated using the full covariance matrix. The cell s.u.'s are taken into account individually in the estimation of s.u.'s in distances, angles and torsion angles; correlations between s.u.'s in cell parameters are only used when they are defined by crystal symmetry. An approximate (isotropic) treatment of cell s.u.'s is used for estimating s.u.'s involving l.s. planes.
Refinement. Refinement of *F*^2^ against ALL reflections. The weighted *R*-factor *wR* and goodness of fit *S* are based on *F*^2^, conventional *R*-factors *R* are based on *F*, with *F* set to zero for negative *F*^2^. The threshold expression of *F*^2^ > 2σ(*F*^2^) is used only for calculating *R*-factors(gt) *etc*. and is not relevant to the choice of reflections for refinement. *R*-factors based on *F*^2^ are statistically about twice as large as those based on *F*, and *R*- factors based on ALL data will be even larger.

### Fractional atomic coordinates and isotropic or equivalent isotropic displacement parameters (Å^2^)

**Table:**

	*x*	*y*	*z*	*U*_iso_*/*U*_eq_	
C1	0.2006 (3)	0.84616 (15)	0.02726 (11)	0.0261 (3)	
H1	0.1077	0.8209	−0.0259	0.031*	
C2	0.3167 (2)	0.87773 (14)	0.09371 (10)	0.0223 (3)	
C3	0.4670 (2)	0.91785 (15)	0.17253 (10)	0.0223 (3)	
H3A	0.6387	0.9299	0.1339	0.027*	
H3B	0.3894	1.0148	0.2026	0.027*	
C4	0.2852 (2)	0.80294 (13)	0.34321 (10)	0.0172 (3)	
C5	0.0548 (2)	0.89754 (13)	0.34541 (10)	0.0189 (3)	
H5	0.0309	0.9688	0.2866	0.023*	
C6	−0.1355 (2)	0.88612 (13)	0.43326 (10)	0.0180 (3)	
H6	−0.2907	0.9504	0.4362	0.022*	
C7	−0.0975 (2)	0.77883 (13)	0.51799 (10)	0.0167 (3)	
C8	0.1301 (2)	0.68501 (13)	0.51207 (10)	0.0164 (3)	
C9	0.3268 (2)	0.69443 (13)	0.42660 (10)	0.0177 (3)	
H9	0.4822	0.6306	0.4246	0.021*	
C10	−0.2529 (2)	0.73992 (13)	0.61999 (10)	0.0173 (3)	
C11	−0.0979 (2)	0.60810 (13)	0.67327 (10)	0.0177 (3)	
H11	−0.1864	0.5181	0.6763	0.021*	
C12	−0.0406 (2)	0.62882 (13)	0.78842 (10)	0.0183 (3)	
C13	0.1469 (2)	0.49897 (14)	0.82127 (11)	0.0237 (3)	
H13A	0.3049	0.4962	0.7693	0.036*	
H13B	0.0765	0.4055	0.8203	0.036*	
H13C	0.1802	0.5115	0.8954	0.036*	
C14	−0.2814 (2)	0.64049 (15)	0.87195 (10)	0.0233 (3)	
H14A	−0.2438	0.6637	0.9432	0.035*	
H14B	−0.3504	0.5455	0.8793	0.035*	
H14C	−0.4052	0.7198	0.8472	0.035*	
O1	0.05931 (15)	0.76695 (9)	0.78783 (7)	0.0193 (2)	
H	0.2022	0.7612	0.7486	0.029*	
O2	0.14038 (15)	0.58334 (9)	0.59873 (7)	0.0187 (2)	
O3	0.48574 (16)	0.80822 (10)	0.26152 (7)	0.0215 (2)	
O4	−0.46595 (15)	0.79629 (10)	0.65811 (7)	0.0215 (2)	

### Atomic displacement parameters (Å^2^)

**Table:**

	*U*^11^	*U*^22^	*U*^33^	*U*^12^	*U*^13^	*U*^23^
C1	0.0272 (7)	0.0300 (7)	0.0206 (7)	−0.0034 (6)	−0.0019 (5)	−0.0033 (5)
C2	0.0241 (7)	0.0221 (6)	0.0176 (6)	−0.0009 (5)	0.0036 (5)	0.0003 (5)
C3	0.0251 (7)	0.0247 (7)	0.0167 (6)	−0.0065 (5)	−0.0002 (5)	0.0016 (5)
C4	0.0184 (6)	0.0194 (6)	0.0144 (6)	−0.0047 (5)	−0.0010 (5)	−0.0043 (5)
C5	0.0222 (6)	0.0188 (6)	0.0161 (6)	−0.0023 (5)	−0.0050 (5)	−0.0006 (5)
C6	0.0173 (6)	0.0185 (6)	0.0187 (6)	−0.0003 (5)	−0.0047 (5)	−0.0033 (5)
C7	0.0163 (6)	0.0180 (6)	0.0166 (6)	−0.0030 (4)	−0.0035 (5)	−0.0038 (5)
C8	0.0204 (6)	0.0151 (6)	0.0150 (6)	−0.0034 (5)	−0.0049 (5)	−0.0024 (4)
C9	0.0171 (6)	0.0186 (6)	0.0175 (6)	−0.0002 (5)	−0.0034 (5)	−0.0038 (5)
C10	0.0166 (6)	0.0197 (6)	0.0174 (6)	−0.0050 (5)	−0.0041 (5)	−0.0041 (5)
C11	0.0161 (6)	0.0187 (6)	0.0176 (6)	−0.0037 (5)	0.0003 (5)	−0.0010 (5)
C12	0.0184 (6)	0.0195 (6)	0.0171 (6)	−0.0046 (5)	−0.0021 (5)	0.0010 (5)
C13	0.0242 (7)	0.0239 (7)	0.0220 (7)	−0.0013 (5)	−0.0043 (5)	0.0033 (5)
C14	0.0214 (7)	0.0302 (7)	0.0175 (6)	−0.0045 (5)	−0.0013 (5)	0.0024 (5)
O1	0.0183 (5)	0.0207 (5)	0.0188 (4)	−0.0044 (3)	−0.0002 (3)	−0.0024 (3)
O2	0.0204 (5)	0.0182 (4)	0.0156 (4)	0.0009 (3)	−0.0002 (3)	0.0006 (3)
O3	0.0197 (5)	0.0272 (5)	0.0155 (4)	−0.0010 (3)	0.0005 (3)	0.0015 (3)
O4	0.0154 (5)	0.0279 (5)	0.0203 (5)	−0.0017 (3)	−0.0014 (3)	−0.0012 (4)

### Geometric parameters (Å, º)

**Table:**

C1—C2	1.187 (2)	C9—H9	0.95
C1—H1	0.95	C10—O4	1.2264 (15)
C2—C3	1.4647 (18)	C10—C11	1.5338 (17)
C3—O3	1.4339 (15)	C11—O2	1.4561 (14)
C3—H3A	0.99	C11—C12	1.5359 (17)
C3—H3B	0.99	C11—H11	1
C4—O3	1.3586 (14)	C12—O1	1.4339 (14)
C4—C9	1.3952 (17)	C12—C14	1.5198 (17)
C4—C5	1.4092 (18)	C12—C13	1.5206 (17)
C5—C6	1.3767 (17)	C13—H13A	0.98
C5—H5	0.95	C13—H13B	0.98
C6—C7	1.3968 (17)	C13—H13C	0.98
C6—H6	0.95	C14—H14A	0.98
C7—C8	1.3906 (17)	C14—H14B	0.98
C7—C10	1.4454 (17)	C14—H14C	0.98
C8—O2	1.3640 (15)	O1—H	0.84
C8—C9	1.3777 (17)		
			
C2—C1—H1	180	C7—C10—C11	105.72 (10)
C1—C2—C3	177.97 (13)	O2—C11—C10	105.17 (9)
O3—C3—C2	112.50 (10)	O2—C11—C12	108.40 (9)
O3—C3—H3A	109.1	C10—C11—C12	116.67 (10)
C2—C3—H3A	109.1	O2—C11—H11	108.8
O3—C3—H3B	109.1	C10—C11—H11	108.8
C2—C3—H3B	109.1	C12—C11—H11	108.8
H3A—C3—H3B	107.8	O1—C12—C14	106.41 (10)
O3—C4—C9	113.90 (10)	O1—C12—C13	110.61 (10)
O3—C4—C5	123.96 (11)	C14—C12—C13	110.22 (10)
C9—C4—C5	122.14 (11)	O1—C12—C11	109.16 (9)
C6—C5—C4	119.68 (11)	C14—C12—C11	110.66 (10)
C6—C5—H5	120.2	C13—C12—C11	109.74 (10)
C4—C5—H5	120.2	C12—C13—H13A	109.5
C5—C6—C7	119.14 (11)	C12—C13—H13B	109.5
C5—C6—H6	120.4	H13A—C13—H13B	109.5
C7—C6—H6	120.4	C12—C13—H13C	109.5
C8—C7—C6	119.71 (11)	H13A—C13—H13C	109.5
C8—C7—C10	107.37 (10)	H13B—C13—H13C	109.5
C6—C7—C10	132.92 (11)	C12—C14—H14A	109.5
O2—C8—C9	123.28 (11)	C12—C14—H14B	109.5
O2—C8—C7	113.74 (10)	H14A—C14—H14B	109.5
C9—C8—C7	122.98 (11)	C12—C14—H14C	109.5
C8—C9—C4	116.33 (11)	H14A—C14—H14C	109.5
C8—C9—H9	121.8	H14B—C14—H14C	109.5
C4—C9—H9	121.8	C12—O1—H	109.5
O4—C10—C7	128.78 (11)	C8—O2—C11	107.95 (9)
O4—C10—C11	125.50 (11)	C4—O3—C3	118.88 (9)
			
O3—C4—C5—C6	−178.39 (10)	O4—C10—C11—O2	−179.31 (10)
C9—C4—C5—C6	1.43 (18)	C7—C10—C11—O2	1.55 (12)
C4—C5—C6—C7	−1.02 (17)	O4—C10—C11—C12	−59.16 (16)
C5—C6—C7—C8	−0.40 (17)	C7—C10—C11—C12	121.70 (11)
C5—C6—C7—C10	178.94 (12)	O2—C11—C12—O1	68.57 (11)
C6—C7—C8—O2	−178.57 (10)	C10—C11—C12—O1	−49.84 (13)
C10—C7—C8—O2	1.93 (14)	O2—C11—C12—C14	−174.64 (9)
C6—C7—C8—C9	1.55 (18)	C10—C11—C12—C14	66.94 (13)
C10—C7—C8—C9	−177.94 (11)	O2—C11—C12—C13	−52.79 (12)
O2—C8—C9—C4	178.99 (10)	C10—C11—C12—C13	−171.21 (10)
C7—C8—C9—C4	−1.15 (18)	C9—C8—O2—C11	178.98 (10)
O3—C4—C9—C8	179.49 (10)	C7—C8—O2—C11	−0.89 (13)
C5—C4—C9—C8	−0.34 (17)	C10—C11—O2—C8	−0.46 (12)
C8—C7—C10—O4	178.84 (12)	C12—C11—O2—C8	−125.94 (10)
C6—C7—C10—O4	−0.6 (2)	C9—C4—O3—C3	−178.56 (10)
C8—C7—C10—C11	−2.06 (12)	C5—C4—O3—C3	1.28 (17)
C6—C7—C10—C11	178.53 (12)	C2—C3—O3—C4	−76.90 (13)

### Hydrogen-bond geometry (Å, º)

**Table:**

*D*—H···*A*	*D*—H	H···*A*	*D*···*A*	*D*—H···*A*
O1—H···O4^i^	0.84	2.01	2.8328 (12)	167
C1—H1···O1^ii^	0.95	2.45	3.3283 (16)	154
C5—H5···O1^iii^	0.95	2.52	3.3809 (15)	152

Symmetry codes: (i) *x*+1, *y*, *z*; (ii) *x*, *y*, *z*−1; (iii) −*x*, −*y*+2, −*z*+1.
